# Strain differences in cuprizone induced demyelination

**DOI:** 10.1186/s13578-017-0181-3

**Published:** 2017-11-03

**Authors:** Qili Yu, Ryan Hui, Jiyoung Park, Yangyang Huang, Alexander W. Kusnecov, Cheryl F. Dreyfus, Renping Zhou

**Affiliations:** 1 Department of Chemical Biology, Ernest Mario School of Pharmacy, Rutgers University, Piscataway, NJ 08854 USA; 2 Department of Neuroscience and Cell Biology, Rutgers Robert Wood Johnson Medical School, Piscataway, NJ 08854 USA; 3 Department of Psychology, School of Arts and Sciences, Rutgers University, Piscataway, NJ 08854 USA; 4 School of Chemical and Environmental Engineering, Wuyi University, Jiangmen, 529020 China; 5 International Healthcare Innovation Institute (Jiangmen), Jiangmen, 529000 China

## Abstract

**Background:**

Multiple sclerosis (MS) is a severe neurological disorder, characterized by demyelination of the central nervous system (CNS), and with a prevalence of greater than 2 million people worldwide. In terms of research in MS pathology, the cuprizone toxicity model is widely used. Here we investigated the contribution of genetic differences in response to cuprizone-induced demyelination in two genetically different mouse strains: CD1 and C57BL/6.

**Results:**

We demonstrate that exposure to a diet containing 0.2% cuprizone resulted in less severe demyelination in the midline of the corpus callosum over the fornix in CD1 mice than C57BL/6 mice. With continuous cuprizone feeding, demyelination in CD1 mice was not prominent until after 7 weeks, in contrast to C57BL/6 mice, which showed prominent demyelination after 4 weeks of exposure. Concomitantly, immunohistochemical analysis demonstrated more oligodendrocytes, as well as fewer oligodendrocyte progenitor cells, microglia and astrocytes in cuprizone treated CD1 mice. We also analyzed 4-weeks-cuprizone treated corpus callosum tissue samples and found that cuprizone treated CD1 mice showed a smaller reduction of myelin-associated glycoprotein (MAG) and a smaller increase of Iba1 and NG2.

**Conclusions:**

These observations suggest that CD1 mice are less vulnerable to cuprizone-induced demyelination than C57BL/6 mice and thus genetic background factors appear to influence the susceptibility to cuprizone-induced demyelination.

**Electronic supplementary material:**

The online version of this article (doi:10.1186/s13578-017-0181-3) contains supplementary material, which is available to authorized users.

## Background

Multiple sclerosis (MS) is a chronic, demyelinating disease in which the myelin sheath, the insulating cover that wraps around axons of neurons, is damaged. The normal function of myelin involves increasing the speed of action potential propagation in axons as well as providing trophic support [1]. Upon damage of myelin, patients suffer from a wide range of symptoms, such as fatigue, pain, spasm, emotional changes, motor deficits and cognitive disorders [2].

The cuprizone neurotoxicity animal model is widely used in MS research [3]. The cuprizone model involves administration of the toxin cuprizone to induce oligodendrocyte apoptosis [4, 5]. Upon treatment with cuprizone, mice typically exhibit profound demyelination in the corpus callosum, the cortex and the superior cerebellar peduncles [4, 5]. In the corpus callosum, apoptosis of oligodendrocytes is observed, together with recruitment of microglia/macrophages, astrocytes and oligodendrocyte progenitor cells (OPCs) [5, 6].

Most investigators use the standard C57BL/6 mouse strain in the cuprizone model to study the different molecular components involved in the complex pathogenic processes leading to corpus callosum demyelination [5]. However, other strains are also used sometimes [7–10]. Thus it is important to know the relative sensitivity of different mouse strains in response to cuprizone toxicity. In our study, we examined the CD1 strain. By comparing it to the C57BL/6 strain, we show that CD1 mice are less vulnerable to cuprizone-induced demyelination in the corpus callosum. Therefore, genetic differences greatly influence the susceptibility of mice to cuprizone-induced damage, and that the influence of mouse strains should be taken into consideration in designing experiments using the cuprizone model.

## Methods

### Mouse strains and cuprizone administration

CD1 mice were purchased directly from Charles River Laboratories (strain code 022) and C57BL/6J mice were purchased from the Jackson Laboratory (stock No. 000664). Both strains of mice were purchased at 8 weeks of age and kept in a pathogen-free facility. Mice were allowed 1 week of acclimation to the environment upon arrival, and then fed with a 0.2% cuprizone-containing diet (Catalog No. TD.01453, Envigo) or a control diet without cuprizone (Catalog No. TD.00217, Envigo). Feeding was ad libitum for a duration ranging from 4 to 7 weeks. For analysis of cuprizone-containing diet intake, both body weights (recorded three times weekly) and food consumption (recorded daily) were closely monitored.

### Tissue sample preparation

**Fig. 1:**
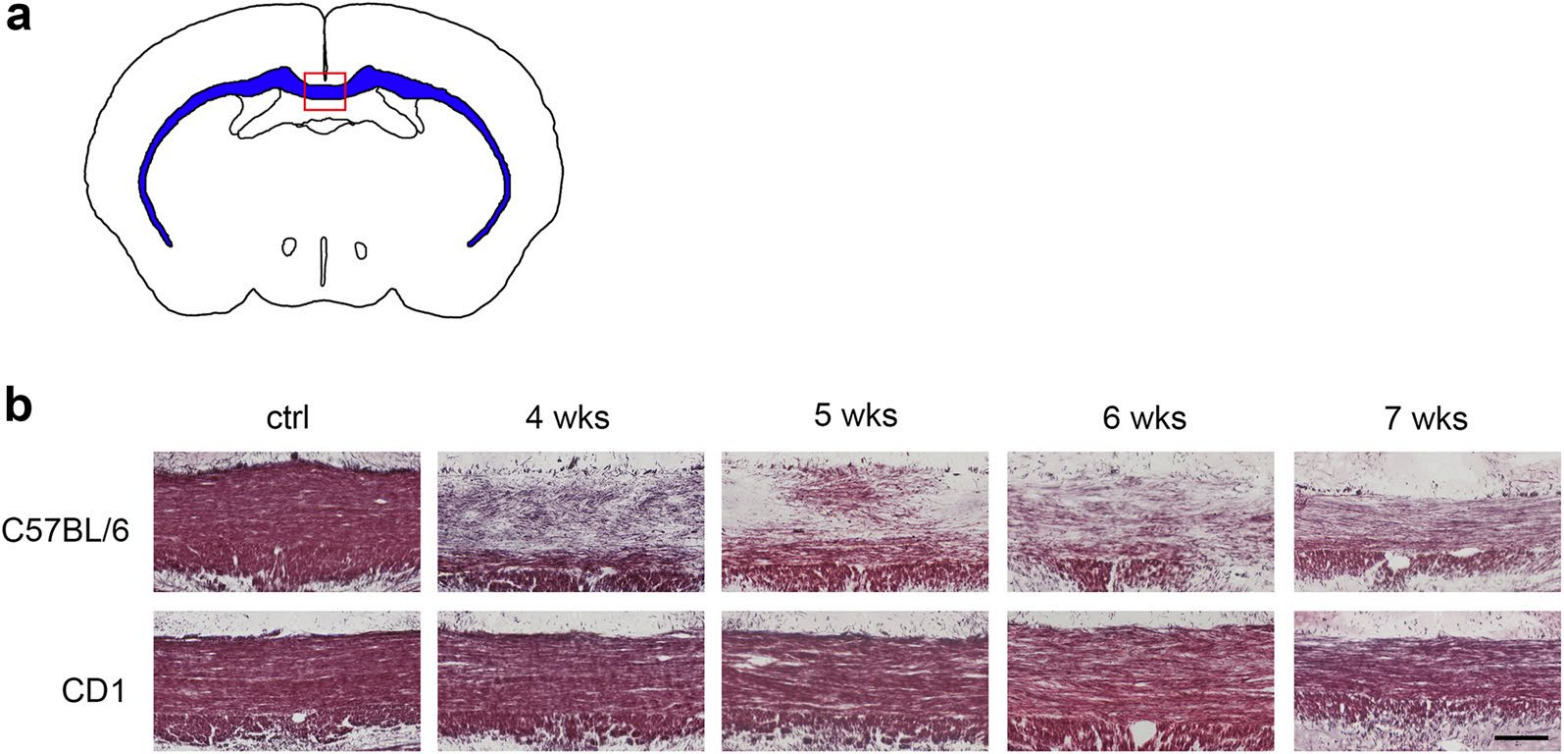
Black gold staining reveals that CD1 mice exhibit much less demyelination relative to C57BL/6 mice following various length of cuprizone exposure. **a** Coronal sections of the corpus callosum were taken above the fornix, approximately between bregma −0.58 mm and bregma −0.82 mm, corresponding to Figs. 36–39 in *The* *Mouse Brain in Stereotaxic Coordinates* [11]. The boxed area depicts the midline of the corpus callosum that was analyzed in B and all the following figures in this study. **b** Representative images of Black gold-stained corpus callosum in C57BL/6 mice and CD1 mice. CD1 mice show relatively intact myelin fibers compared to C57BL/6 mice. Scale bar, 100 µm

For histology and immunohistochemistry, mice were deeply anesthetized by ketamine (Henry Schein, Melville, NY) injection, and transcardially perfused with saline and then with 4% paraformaldehyde (PFA) solution. Mouse brains were then collected and placed in 4% PFA solution for overnight fixation, followed by incubation in 30% sucrose containing phosphate buffered saline (PBS) for cryoprotection. Mouse brains were then frozen in O.C.T. (VWR, Radnor, PA) and cut at 14 μm thickness with a cryostat (Leica, Buffalo Grove, IL). Coronal brain sections were collected focusing on the corpus callosum above the fornix, which is approximately between bregma −0.58 and −0.82 mm (*The Mouse Brain in Sterotaxic Coordinates* [11]) (Fig. 1a). For any of the subsequent analyses, including histology and immunohistochemistry, at least 3 sections were stained per mouse and the average luxol fast blue myelin scoring or cell count/mm^2^ was taken to represent a single mouse.

For western blot analysis, mice were sacrificed by cervical dislocation and mouse brains were collected and placed in a 1 mm brain matrix (Alto Acrylic). A 2 mm section of the corpus callosum tissue overlying the fornix (approximately between bregma 0.14 and − 1.86 mm) was dissected out and frozen at −80 °C. Lysis of the tissue was carried out by homogenizing in diluted NP40 Cell Lysis buffer (ThermoFisher, Bridgewater, NJ) in the presence of protease inhibitor cocktail (1:200, Sigma, St. Louis, MO) and protein concentration was quantified using a BCA protein assay kit (Pierce, Waltham, MA).

### Black gold staining and Luxol fast blue–periodic acid Schiff (LFB–PAS) stain

Black gold stain was carried out using Black-Gold II compound (Histo-Chem, Jefferson, AR) following the manufacturer’s instructions. LFB staining was carried out using LUXOL FAST BLUE—PAS kit (Hitobiotec, Kingsport, TN) following the manufacturer’s instructions followed by PAS counterstaining with a PAS kit (Sigma, St. Louis, MO). Sections were eventually dehydrated with graded ethanol and mounted with permount (Fisher Scientific, Waltham, MA). Images of the midline of the corpus callosum were taken using Virtual Slide microscope 120 (Olympus, Center Valley, PA). For analysis, the sections were scored blind on a scale of 0–3 (Additional file 1: Figure S1) by judging the relative intensity of blue (myelin content) and pink (demyelinated area).

### Immunohistochemistry

For visualization of mature oligodendrocytes, sections were boiled in citrate buffer solution (10 mM, pH 6.0) for antigen retrieval, followed by 1 h blocking in PBS solution containing 10% goat serum and 0.3% Triton and incubated overnight with glutathione S-transferase Pi (GST-π) antibody (Enzo Life Sciences, Farmingdale, NY) at 4 °C. Sections were then rinsed in PBS for washing and further incubated for 3 h in fluorescent secondary antibody (Alexa Fluor Goat anti Rabbit 543, ThermoFisher, 1:200) and to-pro-3 (1:1000, Thermo Fisher, T3605) for nuclear stain.

For visualization of OPCs, sections were blocked with 30% goat serum/0.3% triton in PBS for 1 h, followed by overnight incubation with NG2 antibody (1:750, Millipore, Billerica, MA) at 4 °C. After rinsing in PBS, sections were then incubated with Alexa Fluor goat-anti-rabbit secondary antibody (1:200, Thermo Fisher) together with to-pro-3 (1:1000, Thermo Fisher, T3605) for nuclear stain.

For visualization of microglia/macrophages, sections were blocked with 5% goat serum and 0.3% Triton in PBS for 1 h, followed by overnight incubation at 4 °C in rabbit-anti-Iba1 (1:500, Wako, Richmond, VA). After rinsing in PBS, secondary antibody incubation was carried out using Alexa Fluor goat-anti-rabbit 488 (1:200, Thermo Fisher). To-pro-3 was also added for nuclear stain (1:1000, Thermo Fisher).

For visualization of astrocytes, sections were boiled in citrate buffer solution for antigen retrieval, followed by blocking in 0.1% Triton/2% goat serum containing PBS for 1 h. Glial fibrillary acidic protein (GFAP) antibody (1:200, ThermoFisher) was used as primary antibody for overnight incubation at 4 °C. After rinsing (3 × 5 min) in PBS, secondary antibody incubation was carried out using Alexa Fluor goat-anti-rat 488 (1:400, ThermoFisher) together with to-pro-3 for nuclear stain.

Finally, slides mounted with clear-mount (Electron Microscopy Sciences, Hatfield, PA) were examined and fluorescence images of the midline of the corpus callosum were taken using a three-channel confocal microscope system (Eclipse C1, Nikon, Melville, NY). For all cell counting, positively stained cells are identified by antibody-to-pro-3 colocalization (Additional file 1: Figure S2).

### Western blot

For analysis of myelin basic protein (MBP), myelin-associated glycoprotein (MAG), ionized calcium-binding adapter molecule 1 (Iba1) and Glial fibrillary acidic protein (GFAP) levels, both CD1 and C57BL/6 tissue lysates (6 μg) were combined with 5× Laemmli loading buffer containing 0.1% bromophenol blue, 7.7% Dithiothreitol (DTT), 10% SDS, 50% Glycerol and 60 mM Tris–Cl (pH 6.8). Samples were denatured by boiling and then loaded (6 μg/lane) and analyzed in a 12% acrylamide protein gel. Upon completion of gel electrophoresis, protein was wet transferred onto 0.45 μm nitrocellulose membrane (Bio-Rad), followed by blocking in 5% BSA solution and incubation at 4 °C overnight with primary antibodies against MBP (mouse-anti-MBP, 1:200, Serotec, Hercules, CA), MAG (rabbit-anti-MAG, 1:1000, Santa Cruz, Dallas, TX), Iba1 (rabbit-anti-Iba1, 1:1000, Wako), GFAP (rabbit-anti-GFAP, 1:10000, Abcam, Cambridge, MA) and beta-tubulin (mouse-anti-beta-tubulin, 1:5000, Sigma) for loading control. After washing, fluorescent secondary antibody (Li-cor goat-anti-mouse IRDye 680 for MBP and beta-tubulin and Li-cor goat-anti-rabbit IRDye 800 for Iba1, GFAP and MAG) incubation was performed for 1 h at room temperature, followed by detection of fluorescence signal using odyssey imaging system (Li-cor, Lincoln, NE).

For analysis of NG2 protein levels, 10 μg of protein samples were loaded and run in a 6% acrylamide protein gel. Primary antibody (Rabbit-anti-NG2, 1:1000, Millipore) and secondary antibody (HRP-linked anti-Rabbit IgG, 1:5000, Cell signaling, Danvers, MA) incubation was performed similarly, and detection of chemiluminescence signal was performed using ECL prime reagent (GE healthcare) and GeneGnomeXRQ imaging system (Syngene, Frederick, MD).

### Statistical analysis

Statistical differences between the group data of C57BL/6 and CD1 mice were analyzed using either unpaired student’s t-test or two-way ANOVA analysis where appropriate. Statistical differences between cuprizone treated and control groups within individual strains were analyzed using student’s t-test. Differences were considered to be significant at *P* < 0.05 and data are presented as the mean ± standard error of the mean (SEM).

## Results

The Black gold staining is an easy and widely used approach for visualizing myelin morphology with good resolution of individual myelin fibers [12]. We first performed Black gold staining of the 4 week-cuprizone treated samples, focusing on sections of the corpus callosum above the fornix, which is approximately between bregma −0.58 and −0.82 mm (Fig. 1a). The C57BL/6 mice showed a dramatic loss of myelin staining at this time point (Fig. 1b), which is consistent with previous reports [5]. However, the CD1 mice didn’t show significant myelin loss. This difference in response was also observed at later stages of cuprizone treatment (Fig. 1b).

**Fig. 2:**
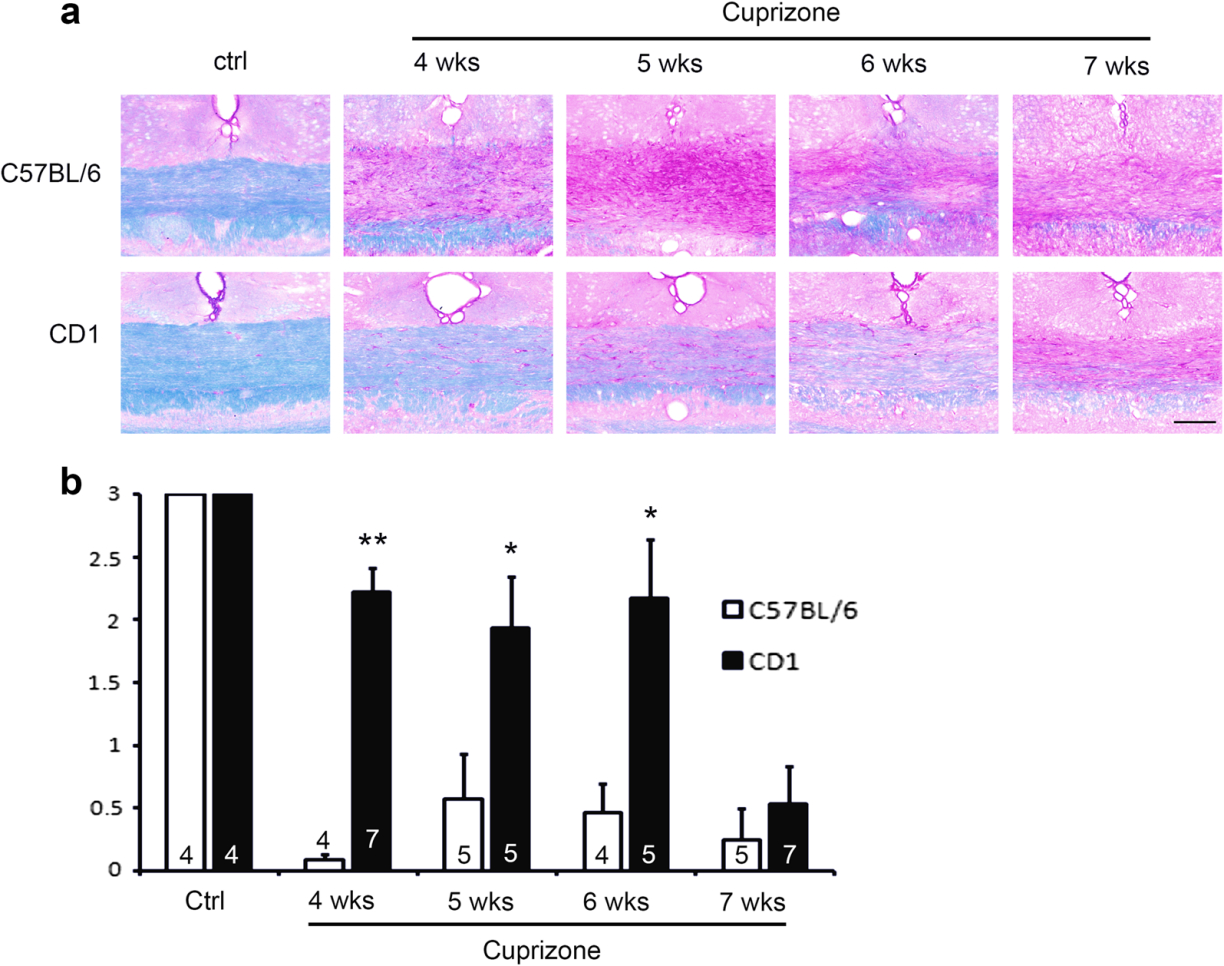
LFB-PAS staining reveals relatively mild demyelination in CD1 mice in comparison to C57BL/6 mice. **a** Representative images of the LFP-PAS stained corpus callosum in C57BL/6 mice and CD1 mice. Myelin shows up as blue stain and the demyelinated area shows up as pink. Note C57BL/6 sections show more pink and less blue compared to CD1 mice. Scale bar, 100 µm. **b** Semi-quantification of demyelination by blind scoring (3 = intact and 0 = complete demyelination). Data is shown as group averages. N = 4 and 7 at 4 weeks for C57 and CD1, respectively. N = 5 and 5 for 5 weeks, N = 4 and 5 for 6 weeks and N = 5 and 7 for 7 weeks. Significant differences between strains were observed at 4 to 6 weeks of cuprizone treatment (student’s t-test). **indicates P < 0.001, *indicates P < 0.05

An alternative histological approach that is complementary to the Black gold staining is the Luxol fast blue–periodic acid Schiff (LFB–PAS) method, which not only stains the myelin (in blue) but also stains the demyelinated areas in pink, thus providing a counter stain to contrast with the positive myelin stain. We thus performed LFB–PAS staining to confirm the results obtained with the Black gold staining (Fig. 2a). The LFB–PAS stained sections were scored blind on a scale of 3 (3 = intact and 0 = full demyelination, Additional file 1: Figure S1). As shown in Fig. 2b, the two strains of mice showed different severity of demyelination at 4–6 weeks of treatment. A close examination of the CD1 corpus callosum at 4–6 weeks of cuprizone exposure shows that CD1 mice do exhibit mild demyelination which is not apparent in the Black gold histological analysis. By 7 weeks, CD1 mice were slightly less demyelinated compared to C57BL/6 mice, but this difference is not statistically significant, although the Black gold data shows CD1 mice still exhibit more Black gold-stained fibers at this point. This slight discrepancy could be due to the different substrates that these two methods detect [13] in that LFB stains phospholipids while Black gold stains myelin proteins. Taken together, the CD1 strain exhibits a delayed and mild demyelination in comparison to C57BL/6 mice.

**Fig. 3:**
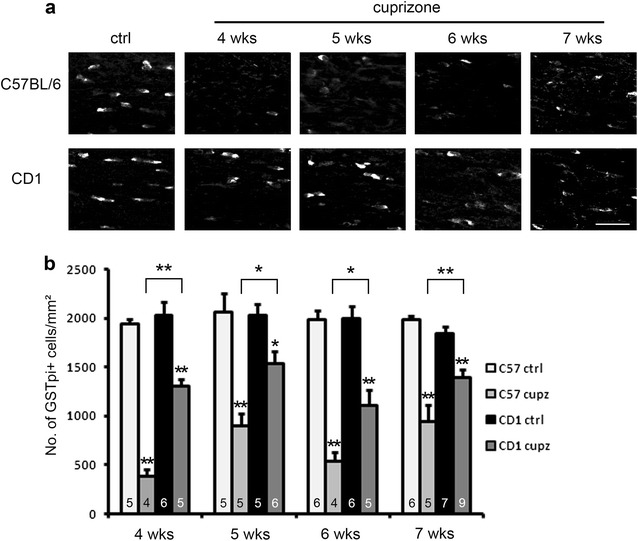
Immunostaining of GSTpi shows relative abundence of mature oligodendrocytes in CD1 mice relative to C57BL/6 mice after various length of cuprizone exposure. **a** Representative images of GSTpi+ mature oligodendrocytes at the midline of the corpus callosum. Scale bar, 30 μm. **b** Quantification of cell density counts. Note CD1 mice and C57BL/6 mice show comparable numbers of mature oligodendrocytes under control conditions. Asterisks above brackets indicate significant differences between strains were observed at all time points of treatment (two way ANOVA analysis for interaction between strain and cuprizone treatment. *P < 0.05; **P < 0.01). Asterisks above the bars indicate significant difference from respective controls (t-test, *P < 0.05; **P < 0.01). Error bars show SEM. N numbers are indicated on individual bars

Because the myelin fibers are formed by oligodendrocytes, we wanted to examine whether the less severe demyelination in CD1 mice is associated with more mature oligodendrocytes being present at the lesion site. We performed immunofluorescence staining using an antibody against GSTpi, a marker for mature oligodendrocytes. As shown in Fig. 3, although both strains fed with cuprizone showed a decrease in GSTpi+ mature oligodendrocytes compared to non-cuprizone-fed controls, the decrease for C57BL/6 mice was more severe than for CD1 mice [significant differences were observed at 4, 5, 6 and 7 weeks of treatment (P < 0.05 in ANOVA at all time points)]. These observations indicate that the lesser demyelination of CD1 mice as shown in the Black gold staining and LFP-PAS stain may be attributed to more oligodendrocytes being present. We also noticed that at 7 weeks of treatment, the number of GSTpi+ oligodendrocytes in the two strains are relatively high, compared to the absence of myelin as shown in the LFB–PAS stain at this time point (compare Figs. 2 and 3 at 7 weeks). This discrepancy could be due to OPCs differentiating into pre-myelinating oligodendrocytes [14] (also see below), which are not myelinating the axons due to continuous cuprizone lesion.

**Fig. 4:**
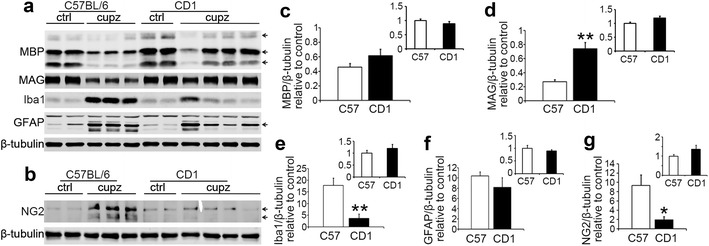
Western blot analysis confirms the relatively mild effect of cuprizone on CD1 mice compared to C57BL/6 following 4 weeks of cuprizone exposure. **a**, **b**, Representative western blot images of MBP, MAG, Iba1, GFAP and NG2 protein. Arrows point to the bands used for quantification of MBP, GFAP and NG2, respectively. **c**–**g**, Statistical analysis of the reduction of MBP, MAG, Iba1, GFAP and NG2. Densitometric data are normalized to β-tubulin and relative to control mice. The decrease in MBP levels are not statistically different in the two strains, while the reduction of MAG level shows significant difference between the two strains (**P < 0.01 in t-test, N = 6 and 8 for C57 and CD1, respectively). The change in Iba1 and NG2 levels after cuprizone treatment are also significantly different between strains (**P < 0.01 in t-test; *P < 0.05 in t-test. N = 6 and 8 for C57 and CD1 mice, respectively). Error bars show SEM. Insets in **c**–**g** show comparison of protein levels of the two strains using 8–9 weeks old unlesioned corpus callosum samples, with data normalized to average of C57BL/6 mice. None of the comparisons show significant difference between the two strains

Since CD1 mice show more myelin and more oligodendrocytes, we wanted to determine whether this difference is reflective of a quantitative difference in the levels of myelin proteins. To this end, we performed western blot analysis using 4 weeks-cuprizone treated corpus callosum samples (Fig. 4). We first examined the levels of myelin basic protein (MBP). As shown in Fig. 4a, c, on average, CD1 mice showed a milder reduction in MBP protein levels after cuprizone treatment compared to C57BL/6 mice, however the difference did not reach statistical significance (P > 0.05 in t-test, N = 6 for C57BL/6 and N = 8 for CD1). A comparison of untreated corpus callosum between CD1 mice and C57BL/6 mice (inset) did not reveal a significant difference (t-test, P > 0.05, N = 6 per group) between strains.

We also analyzed the level of myelin associated glycoprotein (MAG). As shown in Fig. 4a, d, the decrease in MAG level was less in CD1 mice compared to C57BL/6 mice (student’s t-test, P < 0.05, N = 6 for C57BL/6 and N = 8 for CD1), and a comparison of unlesioned corpus callosum sample between the two strains (inset) revealed a similar base line MAG level between the two strains (student’s t-test, P > 0.05, N = 6 per group).

**Fig. 5:**
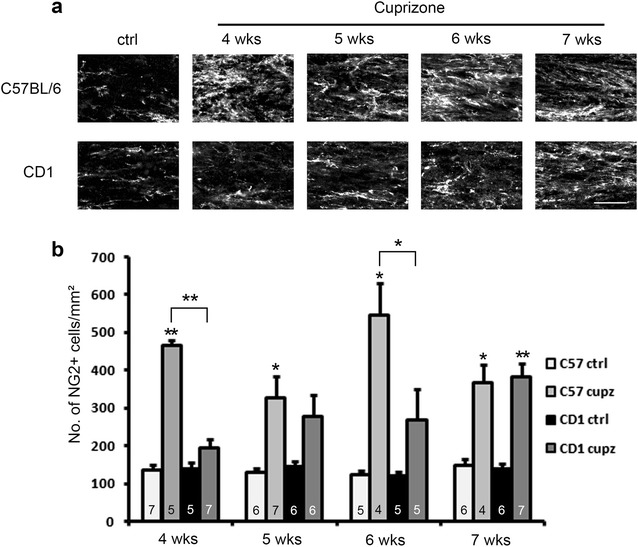
Immunostaining of NG2 shows blunted recruitment of NG2+ oligodendrocyte progenitor cells (OPCs) in CD1 mice relative to C57BL/6 mice at different length of cuprizone exposure. **a** Representative images of NG+ OPCs at the midline of the corpus callosum. Scale bar, 30 μm. **b** Quantification of cell density counts. CD1 mice and C57BL/6 mice show comparable numbers of NG2 and to-pro-3 double positive OPCs under control conditions, and significant differences between strains were observed at 4 and 6 weeks of treatment (asterisks above brackets indicate significant interaction between strain and cuprizone in two-way ANOVA analysis, *P < 0.05; **P < 0.01). At 7 weeks of treatment, the numbers of OPCs in the two strains appear to be similar. Asterisks above the bars indicate significantly different from respective controls (t-test, *P < 0.05; **P < 0.01). Error bars show SEM. N numbers are indicated on individual bars

Because the oligodendrocyte loss induced by the cuprizone lesion is usually accompanied by NG2+ OPC accumulation and differentiation [15], we analyzed the number of NG2 and to-pro-3 double positive OPCs in the cuprizone treated mice (Fig. 5). In C57BL/6 mice, NG2+ OPC number peaked at 4 and 6 weeks, while CD1 mice exhibited a gradual increase in NG2+ cell population, coinciding with increasingly severe demyelination. Although CD1 mice showed a much smaller increase in NG2+ cells at 4 and 6 weeks (P < 0.05 in ANOVA for both 4 and 6 weeks), they do show comparable numbers of NG2+ cells as C57BL/6 strain at 7 weeks. The observation that CD1 mice show less NG2+ OPC recruitment is consistent with the data that CD1 mice show more oligodendrocytes and less demyelination. We also confirmed the morphological data by western blot analysis of NG2 protein level, using 4 weeks-cuprizone treated corpus callosum samples. As shown in Fig. 4b, g, the induction of NG2 protein is significantly lower (P < 0.05, student’s t-test) in CD1 mice, compared to C57BL/6 mice.

**Fig. 6:**
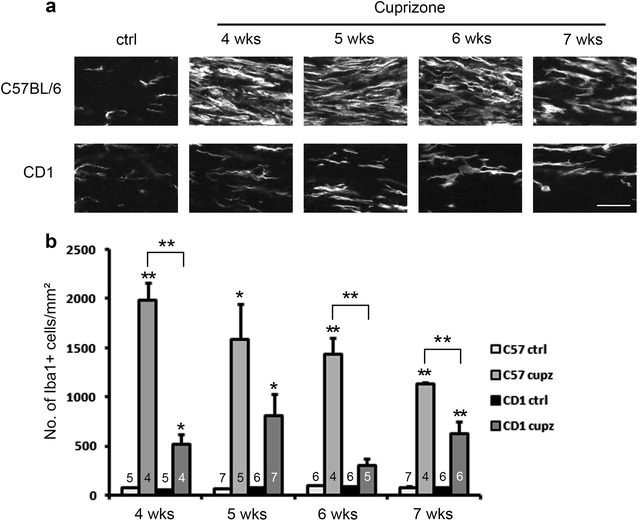
Immunostaining of Iba1 shows blunted recruitment of Iba1+ microglial cells in CD1 mice relative to C57BL/6 mice after various length of cuprizone exposure. **a** Representative images of Iba1+ microglia at the midline of the corpus callosum. Scale bar, 30 μm. **b** Quantification of cell density counts. CD1 mice and C57BL/6 mice show comparable numbers of Iba1 and to-pro-3 double positive microglia under control conditions, and significant differences between strains were observed at 4, 6 and 7 weeks of treatment (asterisks above the brackets indicate significant interaction between strain and cuprizone in two way ANOVA analysis, P < 0.01). Asterisks above the bars indicate significant difference compared to respective controls (t-test, *P < 0.05; **P < 0.01). Error bars show SEM. N numbers are indicated on individual bars

Because cuprizone induced demyelination in the corpus callosum is also known to be associated with microglial/macrophage accumulation and prominent astrogliosis [6], we then analyzed the degree of the innate neuroinflammatory response, focusing on recruitment of microglia to the site of demyelination in the two strains. As the CD1 mice showed less myelin loss following the cuprizone lesion, we expected that CD1 mice would show less microglia compared to C57BL/6 mice. As shown in Fig. 6, the level of Iba1 and to-pro-3 double positive microglia peaked at 4 weeks of cuprizone exposure in C57BL/6 mice, and then gradually decreased in number at 5–7 weeks. In contrast, CD1 mice showed a less prominent microglial response, with the relative increase in microglia being less than C57BL/6 mice at 4, 6 and 7 weeks of cuprizone treatment (P < 0.01 in ANOVA). The numbers of Iba1+ microglia in CD1 mice stayed roughly similar throughout the time points analyzed. A western blot analysis showed that in response to 4 weeks of cuprizone administration, the levels of Iba1 was also lower in CD1 mice compared to C57BL/6 mice (Fig. 4a, e, P < 0.01 in student’s t-test), confirming the morphological observation.

**Fig. 7:**
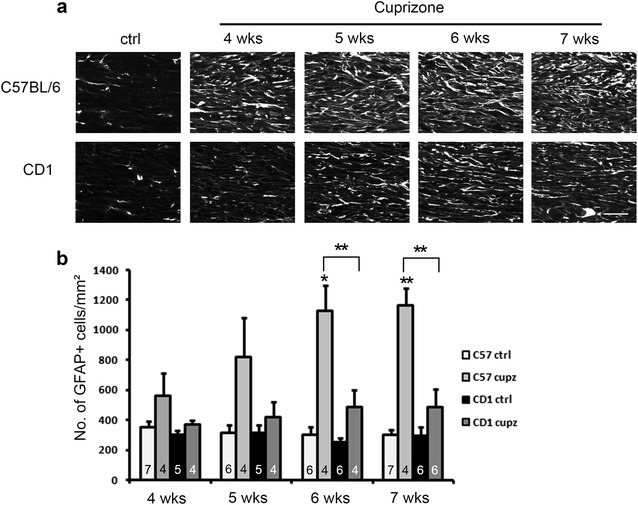
Immunostaining of GFAP shows blunted recruitment of GFAP+ astrocytes in CD1 mice relative to C57BL/6 mice after various length of cuprizone exposure. **a** Representative images of GFAP+ astrocytes at the midline of the corpus callosum. Scale bar, 30 μm. **b** Quantification of cell density counts. CD1 mice and C57BL/6 mice show comparable numbers of GFAP and to-pro-3 double positive astrocytes under control conditions, and significant differences between strains were observed at 6 and 7 weeks of treatment (asterisks above brackets indicate significant interaction between strain and cuprizone in two-way ANOVA analysis, P < 0.01). Asterisks above the bars indicate significant differences from controls (t-test, *P < 0.05; **P < 0.01). Error bars show SEM. N numbers are shown on individual bars

We also analyzed the changes in astrocytes in the cuprizone treated mice. Both strains exhibited a gradual increase in the GFAP and to-pro-3 double positive astrocyte population from 4 to 7 weeks of treatment, with 7 weeks showing the greatest amount of astrogliosis. Compared to C57BL/6 mice, CD1 mice showed on average, a lower degree of astrocyte recruitment at all time points and statistical comparison between strains showed significant difference at 6 and 7 weeks of cuprizone treatment (Fig. 7). Western blot analysis examining GFAP levels in the two strains following 4 weeks of cuprizone treatment showed that CD1 mice exhibited on average a smaller induction of GFAP, which is consistent with the immunohistochemical analysis, but the difference did not reach statistical significance (Fig. 4a, f). Note that the arrow in the figure points to the one band/isoform of GFAP analyzed. Other bands, however, are other GFAP isoforms [16].

**Fig. 8:**
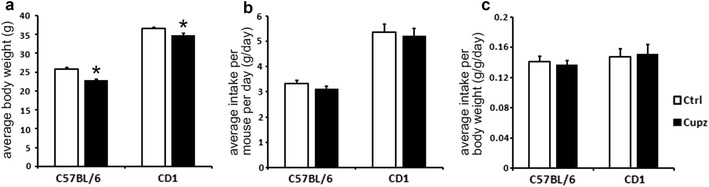
Food intake analysis indicates CD1 mice and C57BL/6 mice exhibit similar amount of cuprizone diet intake per body weight. Although CD1 mice on average exhibit higher body weight compared to C57BL/6 mice (**a**), they also consume more cuprizone containing diet (**b**), as a result, the two strains exhibit similar amount of cuprizone diet intake in terms of grams per body weight (**c**) (No significant differences were seen between groups). Mice were housed in 3–5 animals per cage, and total food intake of individual cages were measured and divided by the number of animals in each individual cage to obtain the average food intake per mouse (**b**), or divided by the total body weight of animals in the cage to obtain the average food intake per body weight (**c**). All values are averaged across 4 consecutive weeks of control diet/cuprizone diet treatment. For **a**, N = 10, 11, 13, 11 respectively. Asterisk indicates significant difference from respective controls (P < 0.01). For **b** and **c**, N = 3 cages per group

Finally, we asked whether the differences in demyelination could be attributed to a difference in cuprizone intake. As shown in Fig. 8, although CD1 mice are on average bigger than C57BL/6 mice (left panel), they also ingest more cuprizone containing diet (middle panel), resulting in a similar amount of food intake relative to body weight (right panel).

## Discussion

We have demonstrated that CD1 mice display less severe demyelination compared to C57BL/6 mice as shown using a variety of parameters. First, histology data of Black gold and LFB-PAS staining clearly indicate that CD1 mice undergo a milder and slower demyelination process. Second, immunohistochemistry data show that CD1 mice exhibit less oligodendrocyte decrease and concomitantly less recruitment of OPCs/microglia/astrocytes following cuprizone intoxication. Third, western blot analysis indicates the reduction of MAG is less in CD1 mice. Fourth, western blot analysis also confirms that CD1 mice show less increase in protein levels of NG2 and Iba1. Lastly, CD1 mice consume a similar amount of cuprizone diet per body weight as compared to C57BL/6 mice.

The earliest studies characterizing cuprizone induced pathology were done using a much higher dosage at 0.5–0.6% [7, 8, 17]. However, the cuprizone induced demyelination in the corpus callosum is best documented in the commonly used C57BL/6 strain and at a dosage of 0.2% [6, 15]. Therefore in our study, we adopted a 0.2% dosage to address cross-strain differences. For C57BL/6 mice, it was observed that the demyelination of C57BL/6 mice is robust at 4–6 weeks of cuprizone treatment, and our findings agree with the previously reported observations. However, recently, there have been several other studies that have addressed the importance of genetic background factors in influencing the extent of demyelination in the cuprizone model. For instance, SJL mice have been shown to be less susceptible to demyelination in the corpus callosum compared to the established C57BL/6 strain [10]. The authors also reported that SJL mice exhibit a unique pattern of demyelination in that the area of the corpus callosum that is immediately lateral to midline seems to demyelinate more prominently than the midline. In our study we observed several cases in which CD1 mice also showed a similar pattern (around 10% of the cuprizone treated CD1 mice, data not shown). In addition to the corpus callosum, demyelination in the cortex induced by cuprizone treatment has also been shown by Skripuletz et al. to be affected by genetic background factors [9]. In their study the authors challenged BALB/cJ mice with 0.2% cuprizone treatment of 6 weeks, and observed that cortical demyelination of BALB/cJ mice was incomplete, as opposed to C57BL/6 strain which shows no detectable cortical myelin at this stage. Taken together, strain differences and genetic background factors greatly influence the sensitivity, the time course and the pattern of cuprizone induced demyelination.

The cuprizone model of induced demyelination has been widely adopted to study the pathological process of MS for the past 20 years. However, the exact mechanism of how cuprizone induces demyelination remains elusive [18]. Nevertheless, a consensus seems to be that cuprizone selectively affects oligodendrocytes. Evidence includes giant mitochondria formation [6, 8, 19–23], increased oxidative stress [18, 19, 24–26] and endoplasmic reticulum (ER) stress [27–29] specifically within oligodendrocytes, eventually leading to oligodendrocyte apoptosis [5, 30]. Importantly this apoptosis precedes the massive demyelination caused by cuprizone administration [18], indicating that oligodendrocyte degeneration is the main cause, as opposed to a secondary immune response. This actually makes the cuprizone model particularly suitable for studying subtypes of MS which show primary oligodendrocyte dystrophy, reminiscent of toxin- or virus-, rather than autoimmunity, induced demyelination [31, 32].

In addition to the direct effect of cuprizone on oligodendrocytes, the involvement of the brain’s resident neuroinflammatory cells such as microglia and astrocyes is also believed to be responsible for the massive demyelination occurring at 4 weeks time point of cuprizone exposure [18]. Important supporting evidence includes (1), a switch from Caspase 3-dependent to Caspase 3-independent form of apoptosis is reported for later stages of cuprizone exposure [33]; (2), cuprizone-induced apoptosis in in vitro oligodendrocyte culture was successful only after addition of pro-inflammatory cytokines, which are produced by microglia and astrocytes [34]; (3), inhibition of microglial activation by minocycline prevented cuprizone induced demyelination in vivo [34]; (4), microglial processes were observed in between myelin lamellae, possibly stripping myelin from axons [8]. In our experiments, we observed a dramatic increase of microglia in C57BL/6 mice following 4 weeks of cuprizone exposure, which is similar to previous reports [6]. However, for the CD1 strain, we did not observe prominent microgliosis (Fig. 6). Even after 7 weeks of treatment, when CD1 mice already show extensive demyelination in the LFB-PAS stain (Fig. 2), the levels of Iba1+ cells are still well below that of the C57BL/6 strain. Thus we propose that the strain differences observed in this study can be at least partially accounted for by a difference in the sensitivity of the microglial cells between the two strains. Future experiments addressing the levels of pro-inflammatory cytokines during the cuprizone treatment period and functional interference with specific cells types such as ablation/inhibition of microglia and/or astrocytes should help elucidate the role of the neuroinflammatory response in contributing to the strain differences observed.

We noted that in our experiment, whereas MAG reduction is significantly less in the CD1 strain (26% reduction in CD1 compared to 73% in C57BL/6), MBP reduction upon cuprizone treatment is not significantly different between the two strains. Given that MAG molecules are localized exclusively in the periaxonal regions of the myelin sheath [35, 36] and that MBP is distributed throughout [37], a relatively abundant MAG level might indicate a relatively intact myelin-axon interface in the CD1 mice, albeit a damaged outer myelin sheath. A similar analysis of myelin protein reduction following cuprizone lesion in other strains that are less vulnerable might be able to address whether this is also the case under other genetic backgrounds.

## Conclusions

In summary, we addressed the important role of genetic background in affecting the demyelination response induced by the neurotoxin cuprizone by comparing CD1 strain versus the commonly used C57BL/6 strain, further extending the current literature on strain differences in the cuprizone model. CD1 mice appear to show relatively mild demyelination and a blunted immune response compared to C57BL/6 mice after the same dosage of cuprizone treatment. Future study of the effects of cuprizone on transcription of myelin proteins, the subcellular morphology of oligodendrocyte and production of growth factors and inflammatory cytokines within CD1 mice might help to elucidate the mechanistic differences underlying strain differences. In turn, this might also help in understanding the wide heterogeneity observed in human MS.

## Additional file

**Additional file 1: Figure S1.** Standard for blind scoring of LFB–PAS stained midline corpus callosum sections. A corresponds to a score of 3 (intact). B corresponds to a score of 2, C corresponds to 1 and D corresponds to 0 (complete demyelination with minimum blue stain of myelin).Scale bar, 100 µm. **Figure S2.** Examples of positively stained cells. A-C, Example images of Iba1+ cells. A, Iba1 fluorescence. B, To-pro-3. C, overlaid image. Arrows point to Iba1+ cells, which show colocalization of Iba1 fluorescence and nuclear to-pro. D-F, same as A-C, but for GFAP staining. Scale bar, 30 μm.

## Abbreviations

CNS: central nervous system
LFB–PAS: Luxol fast blue–periodic acid Schiff
MS: multiple sclerosis
MAG: myelin-associated glycoprotein
MBP: myelin basic protein
mM: millimolar
OPCs: oligodendrocyte progenitor cells
