# Supplementary material for: Strain differences in cuprizone induced demyelination
Source: Cell Biosci. 2017 Nov 3;7:59. doi: 10.1186/s13578-017-0181-3 (PMC5670722; doi:10.1186/s13578-017-0181-3)
Supplement: Supplementary file 1 — Additional file 1: Figure S1. Standard for blind scoring of LFB–PAS stained midline corpus callosum sections. A corresponds to a score of 3 (intact). B corresponds to a score of 2, C corresponds to 1 and D corresponds to 0 (complete demyelination with minimum blue stain of myelin).Scale bar, 100 µm. Figure S2. Examples of positively stained cells. A-C, Example images of Iba1+ cells. A, Iba1 fluorescence. B, To-pro-3. C, overlaid image. Arrows point to Iba1+ cells, which show colocalization of Iba1 fluorescence and nuclear to-pro. D-F, same as A-C, but for GFAP staining. Scale bar, 30 μm. [file 13578_2017_181_MOESM1_ESM.pdf]

Additional file 1

Strain differences in cuprizone induced demyelination

Qili Yu[1], Ryan Hui[1],Jiyoung Park[1], Yangyang Huang[2], Alexander W. Kusnecov[3], Cheryl F. Dreyfus[2]&

Renping Zhou[1,4,5]

[1]Department of Chemical Biology, Ernest Mario School of Pharmacy, Rutgers University. Piscataway, NJ 08854 USA

[2]Department of Neuroscience and Cell Biology, Rutgers Robert Wood Johnson Medical School. Piscataway, NJ 08854 USA

[3]Department of Psychology, School of Arts and Sciences, Rutgers University. Piscataway, NJ 08854 USA

[4]School of Chemical and Environmental Engineering, Wuyi University, Jiangmen 529020 China

[5]International Healthcare Innovation Institute (Jiangmen), Jiangmen 529000 China

Email addresses:

Q.Y., yuql18@gmail.com

R.H., ryan.p.hui@gmail.com

J.P., j.park0527@gmail.com

Y.H., huangy4@gmail.com

A.W.K., kusnecov@psych.rutgers.edu

C.F.D, dreyfus@rwjms.rutgers.edu

R.Z., rzhou@pharmacy.rutgers.edu

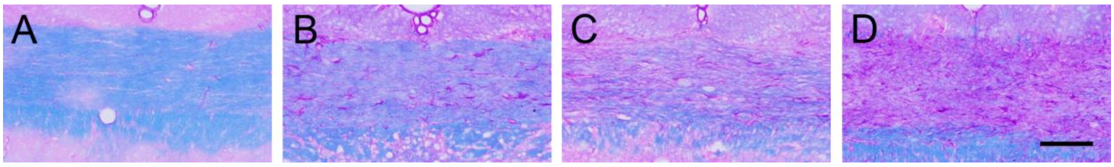

Additional file 1: Figure S1. Standard for blind scoring of LFB-PAS stained midline corpus callosum sections. A corresponds to a score of 3 (intact). B corresponds to a score of 2, C corresponds to 1 and D corresponds to 0 (complete demyelination with minimum blue stain of myelin). Scale bar, 100 µm.

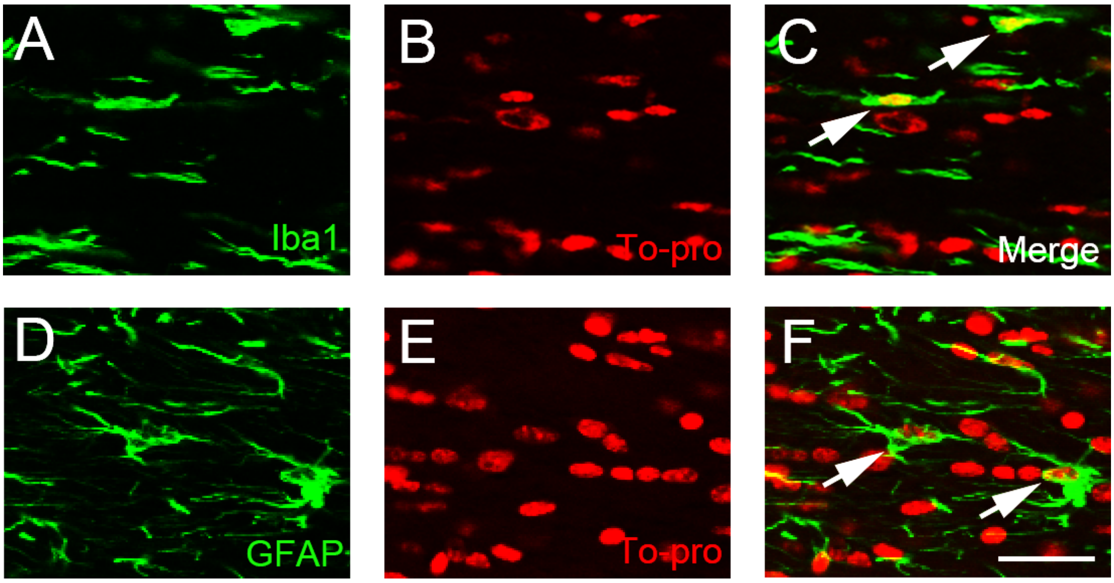

Additional file 1: Figure S2. Examples of positively stained cells. A-C, Example images of Iba1+ cells. A, Iba1 fluorescence. B, To-pro-3. C, overlaid image. Arrows point to Iba1+ cells, which show colocalization of Iba1 fluorescence and nuclear to-pro. D-F, same as A-C, but for GFAP staining. Scale bar, 30 µm.
